# Association between early nutrition support and 28-day mortality in critically ill patients: the FRANS prospective nutrition cohort study

**DOI:** 10.1186/s13054-022-04298-1

**Published:** 2023-01-07

**Authors:** Emmanuel Pardo, Thomas Lescot, Jean-Charles Preiser, Pablo Massanet, Antoine Pons, Samir Jaber, Vincent Fraipont, Eric Levesque, Carole Ichai, Laurent Petit, Fabienne Tamion, Garry Taverny, Priscilla Boizeau, Corinne Alberti, Jean-Michel Constantin, Marie-Pierre Bonnet, the FRANS study group, Désiré Samba, Jean-Denis Moyer, Philippe Montravers, Nicolas Mongardon, Arnaud Meffert, Audrey De Jong, Fouad Belafia, Jérome Morel, Karim Asehnoune, Pierre-Joachim Mahé, Alain D’Hondt, Nicolas Paquot, Marc Leone, Michel Kaidomar, Ludovic Grech, Eliane Gouteix, Elise Barsam, Jacques Duranteau, Orianne Martinez

**Affiliations:** 1 Sorbonne Université, GRC 29, AP-HP, DMU DREAM, Département d’Anesthésie-Réanimation, Hôpital Saint-Antoine, Assistance publique-hôpitaux de Paris, 184 Rue du Faubourg Saint-Antoine, 75012 Paris, France; 2 Service des Soins intensifs, Hôpital Erasme, Université Libre de Bruxelles, Brussels, Belgium; 3 Département Anesthésie-Réanimation, Centre Hospitalier Universitaire Nîmes, 30000 Nîmes, France; 4 Sorbonne Université, GRC 29, AP-HP, DMU DREAM, Département d’Anesthésie-Réanimation, Hôpital Pitié-Salpêtrière, Assistance publique-hôpitaux de Paris, 75013 Paris, France; 5 Department of Anaesthesia and Intensive Care Unit, Regional University Hospital of Montpellier, St-Eloi Hospital, University of Montpellier. PhyMedExp, INSERM U1046, CNRS UMR, 9214 Montpellier Cedex 5, France; 6 Service de Soins Intensifs, Centre Hospitalier Régional de Liège, 4000 Liège, Citadelle, Belgium; 7 Service d’anesthésie-réanimation chirurgicale, GHU Henri-Mondor, 94000 Créteil, France; 8 Université Côte d’Azur, Centre Hospitalier Universitaire de Nice, Département Anesthésie-Réanimation, Nice, France; 9 Service de réanimation chirurgicale et traumatologique Pellegrin place Amélie Raba-Léon, 33000 Bordeaux, France; 10 Service de Médecine Intensive Réanimation, CHU Rouen, Université de Normandie, UNIROUEN, INSERM U1096, 76000 Rouen, France; 11 AP-HP, Hôpital Robert-Debré, Unité d’Epidémiologie Clinique, 48 bd Serurier, 75019 Paris, France; 12 Sorbonne Université, Département Anesthésie-Réanimation, Hôpital Armand Trousseau, DMU DREAM, GRC 29, AP-HP, Paris, France; 13 Université Paris Cité, INSERM, INRA, Centre for Epidemiology and Statistics Sorbonne Paris Cité (CRESS), Obstetrical Perinatal and Pediatric Epidemiology Research Team, EPOPé, Maternité Port Royal, 53 avenue de l’Observatoire, 75014 Paris, France

**Keywords:** Clinical nutrition, Intensive care unit, Enteral nutrition, Parenteral nutrition, Critical illness, Clinical nutrition guidelines, Mortality, Early nutrition support

## Abstract

**Background:**

Current guidelines suggest the introduction of early nutrition support within the first 48 h of admission to the intensive care unit (ICU) for patients who cannot eat. In that context, we aimed to describe nutrition practices in the ICU and study the association between the introduction of early nutrition support (< 48 h) in the ICU and patient mortality at day 28 (D28) using data from a multicentre prospective cohort.

**Methods:**

The ‘French-Speaking ICU Nutritional Survey’ (FRANS) study was conducted in 26 ICUs in France and Belgium over 3 months in 2015. Adult patients with a predicted ICU length of stay > 3 days were consecutively included and followed for 10 days. Their mortality was assessed at D28. We investigated the association between early nutrition (< 48 h) and mortality at D28 using univariate and multivariate propensity-score-weighted logistic regression analyses.

**Results:**

During the study period, 1206 patients were included. Early nutrition support was administered to 718 patients (59.5%), with 504 patients receiving enteral nutrition and 214 parenteral nutrition. Early nutrition was more frequently prescribed in the presence of multiple organ failure and less frequently in overweight and obese patients. Early nutrition was significantly associated with D28 mortality in the univariate analysis (crude odds ratio (OR) 1.69, 95% confidence interval (CI) 1.23–2.34) and propensity-weighted multivariate analysis (adjusted OR (aOR) 1.05, 95% CI 1.00–1.10). In subgroup analyses, this association was stronger in patients ≤ 65 years and with SOFA scores ≤ 8. Compared with no early nutrition, a significant association was found of D28 mortality with early enteral (aOR 1.06, 95% CI 1.01–1.11) but not early parenteral nutrition (aOR 1.04, 95% CI 0.98–1.11).

**Conclusions:**

In this prospective cohort study, early nutrition support in the ICU was significantly associated with increased mortality at D28, particularly in younger patients with less severe disease. Compared to no early nutrition, only early enteral nutrition appeared to be associated with increased mortality. Such findings are in contrast with current guidelines on the provision of early nutrition support in the ICU and may challenge our current practices, particularly concerning patients at low nutrition risk.

*Trial registration* ClinicalTrials.gov Identifier: NCT02599948. Retrospectively registered on November 5th 2015.

**Supplementary Information:**

The online version contains supplementary material available at 10.1186/s13054-022-04298-1.

## Background

Patients admitted to the ICU suffer from acute critical illness. This induces major catabolic stress, which may result in severe muscle wasting and prolonged impaired functional outcomes [1]. It is commonly accepted that providing adequate nutrition with essential nutrients may help to attenuate the consequences of the catabolic response. However, identifying the appropriate timing, amount and route of nutrition support remains a complex challenge for ICU physicians. Nutritional therapy limits the risk of an energy or protein deficit, which was associated in previous retrospective studies with poor outcomes, such as prolonged ICU and hospital stays, prolonged mechanical ventilation durations and higher incidences of infectious complications [2–4]. The risk of complications is even higher in patients identified with high nutrition risk at ICU admission [5]. However, recent randomised control trials (RCTs) and meta-analyses failed to demonstrate any benefit of the nutritional guideline target adequacy during the first weeks of ICU on the outcomes studied [6–12].

International guidelines recommend the early introduction of hypocaloric enteral nutrition within the first 48 h, except in cases of uncontrolled shock, hypoxemia or acidosis [13–15]. The rationale of these recommendations is based on the results of a meta-analysis reporting the benefit of early enteral nutrition for decreasing the incidence of infectious complications; however, this positive effect was not found when studies involving non-critically ill patients were excluded [14]. The most recent meta-analysis, conducted by the Cochrane group, including seven RCTs published between 1993 and 2012, also reported ‘very low-quality evidence’ in favour of early over delayed enteral nutrition in ICU patients [16]. Furthermore, early initiation with rapid achievement of the energy target has not shown a significant benefit and may even cause harm [12, 17]. In addition, in a recent study by Ortiz‐Reyes et al., early enteral nutrition showed no benefit compared to delayed enteral in ventilated patients receiving vasopressor or inotropic therapies after adjusting for the illness severity [18]. Finally, some concerns have recently emerged about the possible increased risk of digestive complications such as mesenteric ischaemia associated with early enteral feeding [19, 20].

Nutritional practices in the ICU may significantly differ between units as well as between patients. Current prescriptions in the ICU setting and factors influencing caregivers in their choices remain poorly described. We set out to analyse data from a real-life, prospective, multicentric cohort study, to explore the impact of early nutrition, and its route of delivery, on patient mortality. Specifically, the purposes of this observational study were to (1) describe current practices and factors associated with the prescription of early nutrition support within the first 48 h in the ICU, and (2) conduct an adjusted analysis of the association between early nutrition support and 28-day mortality.

## Methods

### Study design

We performed a multicentre, prospective, observational study specifically designed to explore nutrition practices for critically ill patients during the first 10 days of ICU stay (from day 1 to day 10, with D0 corresponding to the day of ICU admission), the ‘French-Speaking ICU Nutritional Survey’ (FRANS) study. This study was conducted in 23 ICUs in France and three in Belgium. Patients were included over 3 consecutive months, from February to June 2015 for French ICUs and from May to August 2015 for Belgian ICUs. The patients were followed for 28 days. The ethical committee of each institution approved the FRANS study and the trial was retrospectively registered on ClinicalTrials.gov under the reference NCT02599948. Reporting of this study was in accordance with the Strengthening the Reporting of Observational Studies in Epidemiology (STROBE) statement and guidelines [21].

### Study population

Critically ill adult patients with an expected length of stay greater than 3 days in the ICU were included in the FRANS study. The exclusion criteria were as follows: aged under 18 years, the patient or next-of-kin’s refusal, a prior medical decision to limit or discontinue life-sustaining therapies. The study protocol allowed for secondary exclusion after a patient’s inclusion in the study if a decision was made to limit life-sustaining therapy within the first 10 days of their ICU stay.

### Data collection

In each participating ICU, a referring physician was responsible for data collection. Data were prospectively collected from medical charts and daily prescriptions using a dedicated case report form.

At the baseline, the following data were recorded: patient demographic characteristics (age, sex, height, weight), admission diagnosis (medical or surgical), Simplified Acute Physiology Score (SAPS) II and use of mechanical ventilation [22]. Organ dysfunction was evaluated with the Sequential Organ Failure Assessment (SOFA) score and the severity of illness with the Acute Physiology and Chronic Health Evaluation (APACHE II) score [23, 24]. Patient weight was measured at ICU admission by weighted beds or Hoyer lift with integrated weighting system. Admission actual weight was used to present intakes in kcal/kg/day and to calculate adjusted body weight for obese patients. Underweight was defined by a body mass index (BMI) < 18 kg/m^2^, overweight by a BMI between 25 and 30 kg/m^2^ and obesity by a BMI > 30 kg/m^2^.

The following nutritional data were collected daily during the 10 first days of ICU stay: volume (mL/day) and route of administration of nutrition support (enteral, parenteral or both), volume of propofol infusion (mL/day) and intravenous glucose (mL/day), prescription of vitamin therapy and trace elements (type and volume in mL). We then calculated the patients’ total energy and protein intakes per kilogram of body weight received by patients, from both nutritional and non-nutritional solutions. Non-nutritional calories were calculated from both the daily propofol and glucose intakes. Propofol accounted for 1.1 kcal/ml and dextrose for 4 kcal/g. Calculated non-nutritional energy was added to energy received through enteral and/or parenteral nutrition and presented as ‘total caloric intake.’ Regarding obese patients, the nutritional intake per kilogram was based on their adjusted body weight (BW) (ideal BW + 0.25 × (actual BW − ideal BW) [25]. The ideal BW was based on the patient's height at a BMI of 25 kg/m^2^. We considered that patients reached the recommended energy and protein targets if their intakes were above 25 kcal/kg/day and 1.3 g/kg/day, respectively [14]. To consider the progressive rise in energy and protein intakes, instead of using an average intake smoothed over the follow-up, patients were considered to have reached the target if the total daily intake observed during the nutritional follow-up was above the guideline threshold at least once. Early nutrition support was defined as the administration of any nutritional solution, enteral and/or parenteral, during the first 48 h after ICU admission, as described in international guidelines [14, 15].

Specific information concerning organ support and critical care therapies was collected daily: invasive mechanical ventilation, use of neuromuscular blocking agents, vasopressors and sedation. Digestive tract events were also noted every day, including bowel movements, emesis and diarrhoea. Feeding intolerance was defined as the occurrence of emesis and/or diarrhoea concomitant with enteral nutrition administration.

The collected patient outcomes were the total duration of both invasive and noninvasive mechanical ventilation, length of ICU stay, ICU mortality and day-28 (D28) mortality.

### Statistical analysis

We first described the demographic characteristics, use of organ support and patient outcomes in the study population. We then studied the nutritional intake received during the nutritional follow-up and the incidence of digestive complications or feeding intolerance. For patients discharged from the ICU, lost to follow-up or who died within the first 10 days of the ICU stay, nutritional data were analysed for the available days. Energy and protein mean intakes were calculated considering the number of days of follow-up. Missing data were marginal in our cohort (< 5%).

Then, we explored factors associated with the prescription of early nutrition support using univariate and multivariable analyses. Early nutrition was studied first as a binary variable (yes/no) and then in three categories (early enteral (EN), early parenteral (PN) and no early nutrition). As patients treated with mixed early nutrition (parenteral and enteral) had the same demographic and nutritional characteristics as those treated with early parenteral alone, they were included in the early parenteral group. The covariates included in the models were selected based on an a priori hypothesis according to the literature and the univariate analysis. For the binary variable (yes/no), we used a multivariable logistic model that included the following factors: age, sex, admission diagnosis, BMI and admission SOFA score. When early nutrition was explored in three categories (EN/PN/none), we used a multinomial, multivariable logistic regression analysis that included the following variables: age, sex, admission diagnosis, early invasive ventilation and early vasopressors. Separate organ support was chosen in this model to assess the specific influence of invasive mechanical ventilation on the choice of nutrition route. The reference category was ‘no early nutrition support.’ Results are reported as crude (ORs) and adjusted odds ratios (aORs) with a 95% confidence interval (95% CI). Multivariable model selection was performed using a two-way stepwise procedure with the aim of minimising the Akaike information criterion (AIC). We assessed multicollinearity between variables by computing the variance inflation factor (VIF) and using the Farrar–Glauber test. The goodness of fit was studied using the Hosmer–Lemeshow test.

Finally, we explored the association between early nutrition support (binary and in three categories) and 28-day mortality with univariable logistic regression and multivariable, multilevel analysis with a random effect on the ICU that admitted the patient (patient in the first level and centre of inclusion in the second). To rule out indication bias concerning early nutrition support, multivariable analyses were also performed using a propensity score. The propensity to belong to the early nutrition group was modelled by the nonparametric gradient boosting machine learning algorithm included in the Twang package [26]. Confounding factors included in the propensity score were age, sex, admission diagnosis, BMI and SOFA score at admission. The balance of the propensity model was assessed by the standardised effect size of the variables (Additional file 1: Fig. S1). Standardised effects of less than 0.20 were considered low (better balance), 0.40 as moderate and 0.60 as large. The weights calculated from the propensity score were used for the weighting of the multivariable logistic regression. In the weighted population, it was possible to assess the association with 28-day mortality in a pseudo-population in which the characteristics of subjects receiving or not receiving early nutrition were balanced. We chose to use the double robust approach to lower the risk of bias relative to the distribution difference of studied cofactors, which may persist even after propensity score weighting [27]. Accordingly, we adjusted our propensity-weighted (PW) regression model for all covariates included in the propensity score model.

Subgroup analyses were performed to assess the strength of the association between early nutrition support and 28-day mortality in specific populations: male/female, age ≤ or > 65 y.o., medical or surgical admission diagnosis, BMI range (underweight, standard, overweight, obese) and admission SOFA score ≤ or > 8. We modified the multivariable multilevel logistic regression analysis in each subgroup analysis by removing the respective subgroup variable. These results are presented as a forest plot.

Variables were compared between groups by Fisher’s exact and Chi-squared tests for categorical variables, and by a Mann–Whitney or Student’s t-test according to the normality of quantitative variables, as assessed by a Shapiro–Wilk test. Comparisons of more than two groups were conducted with one-way ANOVA or the Kruskal–Wallis test. The tests were two-sided, with an alpha risk *α* = 0.05. Results are given as the median (25th–75th percentiles) or mean (standard deviation) for quantitative variables, as appropriate, and the number of patients (with percentage proportion) for qualitative variables. A P-value less than 0.05 was considered statistically significant. R software (version 4.1.2, GUI 1.77 for Macintosh, GNU and GPL licences, The R Project for Statistical Computing, Vienna, Austria) and the RStudio interface (version 2022.02.0, Boston, MA, USA) were used to perform the statistical analyses.

## Results

### Characteristics of the study population and nutrition management

**Fig. 1:**
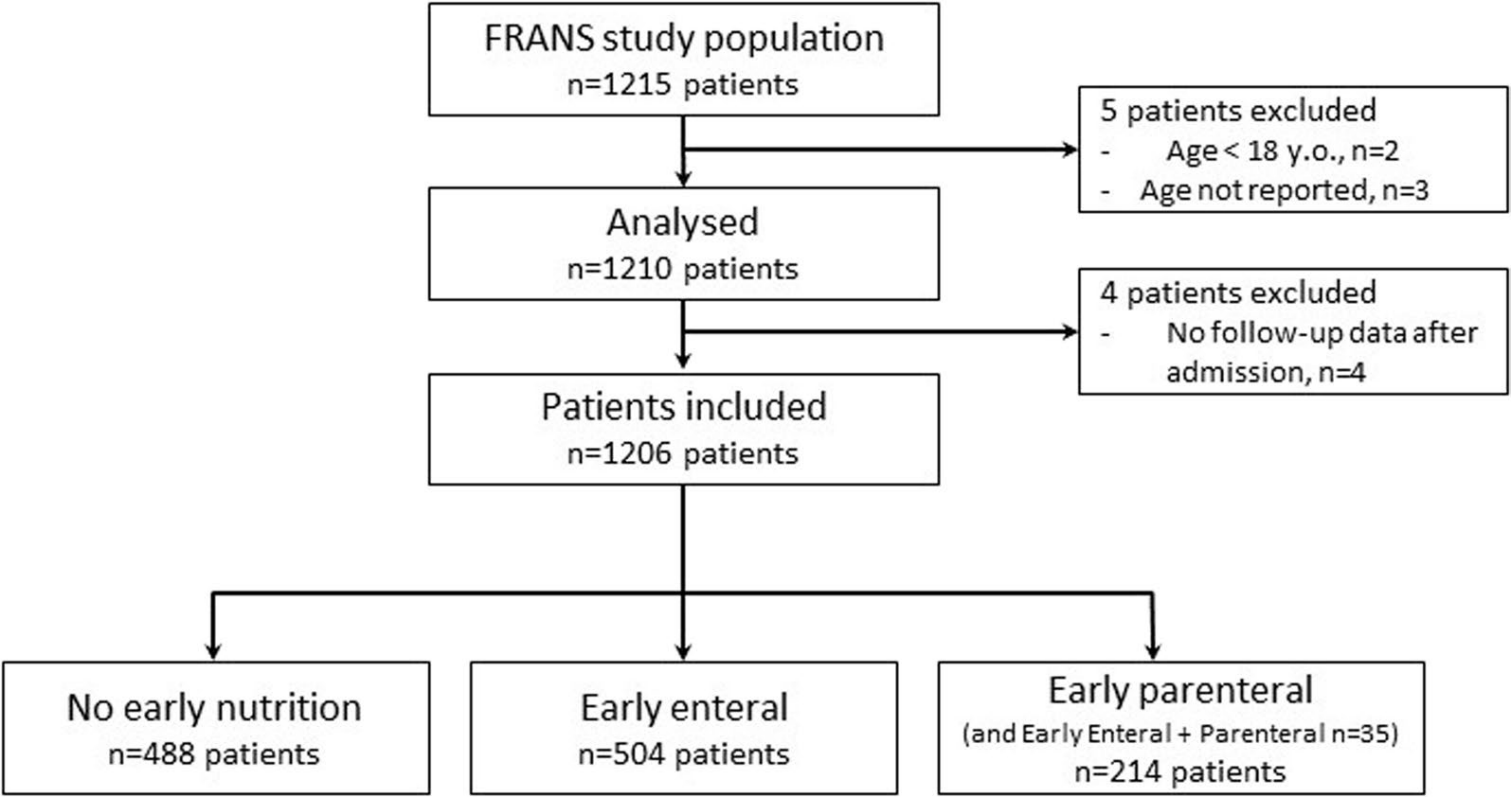
Flowchart of inclusion and early-nutrition-type distribution

Overall, 1206 patients were included in the present study (Fig. 1). Invasive mechanical ventilation was used for 979 (81.2%) patients for a median duration of 7 [3–15] days. The median ICU length of stay was 10 [6–20] days. The overall ICU mortality was 18.5% (*n* = 223), and the D28 mortality was 18.8% (*n* = 226) (Table 1). Our hospital mortality prediction based on the admission SAPS II score was 32%.

**Table 1:** Characteristics of the study population, nutrition management and patients’ overall outcomes according to the 28-day mortality

	Overall (*n* = 1206)	Survivors (*n* = 975)	Non-survivors (*n* = 226)	*P* value
*Patient admission characteristics*				
Age (years)	62.9 [51.2, 72.8]	61.6 [50.3, 71.4]	66.5 [58.1, 76.6]	< 0.001
Height (cm)	170.0 [165.0, 176.0]	170.0 [164.0, 176.0]	170.0 [165.0, 175.0]	0.712
Weight (kg)	75.0 [65.0, 87.0]	75.0 [65.0, 87.0]	75.0 [63.0, 85.0]	0.174
Sex (%)				
Female	393 (32.6)	324 (33.2)	67 (29.6)	0.338
Male	813 (67.4)	651 (66.8)	159 (70.4)	
Admission type (%)				
Surgical	603 (50.0)	535 (54.9)	65 (28.8)	< 0.001
Medical	603 (50.0)	440 (45.1)	161 (71.2)	
BMI (kg/m^2^)	25.7 [22.7, 29.7]	25.7 [22.8, 29.8]	25.3 [22.7, 28.6]	0.123
BMI range (%)				
< 18	37 (3.2)	27 (2.9)	10 (4.7)	0.207
18–25	477 (41.1)	385 (40.9)	91 (42.5)	
25–30	369 (31.8)	295 (31.3)	72 (33.6)	
> 30	277 (23.9)	234 (24.9)	41 (19.2)	
Country (%)				
France	1003 (83.2)	817 (83.8)	182 (80.5)	0.279
Belgium	203 (16.8)	158 (16.2)	44 (19.5)	
University hospital (%)				
Yes	1060 (87.9)	870 (89.2)	187 (82.7)	0.010
No	146 (12.1)	105 (10.8)	39 (17.3)	
*Severity and organ support*				
Admission SAPS II score	44.0 [33.0, 57.0]	42.0 [32.0, 54.0]	53.0 [41.8, 65.3]	< 0.001
Admission SOFA score	8.0 [5.0, 11.0]	8.0 [4.0, 10.0]	9.0 [7.0, 12.0]	< 0.001
Admission APACHE II score	19.0 [13.0, 24.0]	18.0 [13.0, 23.0]	22.0 [17.0, 28.0]	< 0.001
Early vasopressors (%)	754 (62.6)	572 (58.8)	180 (79.6)	< 0.001
Early IMV (%)	870 (72.3)	684 (70.3)	182 (80.9)	0.002
Sedation (%)	844 (70.0)	649 (66.6)	191 (84.5)	< 0.001
NMBA (%)	216 (17.9)	156 (16.0)	59 (26.1)	0.001
Vasopressors (%)	818 (67.8)	615 (63.1)	200 (88.5)	< 0.001
IMV (%)	979 (81.2)	776 (79.6)	200 (88.5)	0.003
NIMV (%)	408 (33.8)	347 (35.6)	60 (26.5)	0.012
*Early nutritional intake*				
Timing of enteral after admission (hours)	37.6 [24.2, 64.6]	37.1 [24.2, 63.8]	39.9 [24.5, 72.2]	0.331
Early nutrition (%)				
None	488 (40.5)	416 (42.7)	71 (31.4)	0.020
EN	504 (41.8)	392 (40.2)	110 (48.7)	
EN + PN	35 (2.9)	26 (2.7)	8 (3.5)	
PN	179 (14.8)	141 (14.5)	37 (16.4)	
Total early caloric intake (kcal/kg/day)	10.03 [4.81, 18.09]	9.77 [4.59, 17.39]	11.37 [6.25, 20.53]	0.006
Early non-nutritional calories (kcal/kg/day)	3.16 [1.25, 5.63]	3.13 [1.25, 5.56]	3.23 [1.29, 6.08]	0.471
Early protein (g/kg/day)	0.24 [0.00, 0.64]	0.21 [0.00, 0.63]	0.37 [0.00, 0.72]	0.005
*10-day nutritional intake and adverse effects*				
Enteral nutrition (%)	753 (62.4)	579 (59.4)	170 (75.2)	< 0.001
Parenteral nutrition (%)	406 (33.7)	323 (33.1)	80 (35.4)	0.567
Total Caloric intake (kcal/kg/day)	16.94 [7.18, 22.82]	16.08 [6.17, 22.59]	18.43 [11.85, 23.89]	< 0.001
Non-nutritional calories (kcal/kg/day)	2.16 [1.08, 3.92]	2.11 [1.06, 3.85]	2.53 [1.21, 4.28]	0.033
Protein intake (g/kg/day)	0.62 [0.17, 0.91]	0.59 [0.09, 0.90]	0.67 [0.38, 0.98]	0.002
Diarrhoea (%)	322 (26.7)	262 (26.9)	59 (26.1)	0.880
Bowel movement (%)	969 (80.3)	797 (81.7)	168 (74.3)	0.015
Emesis (%)	186 (15.4)	144 (14.8)	40 (17.7)	0.318
Feeding intolerance (%)	257 (34.0)	195 (33.7)	59 (34.7)	0.876

Results are presented as the median (25th–75th percentiles) for quantitative variables and patient number (column proportion) for qualitative variables

*BMI* body mass index, *SAPS* Simplified Acute Physiology Score, *SOFA* Sequential Organ Failure Assessment, *APACHE* Acute Physiology and Chronic Health Evaluation, *NMBA* neuromuscular blocking agents, *IMV* invasive mechanical ventilation, *NIMV* noninvasive mechanical ventilation

**Fig. 2:**
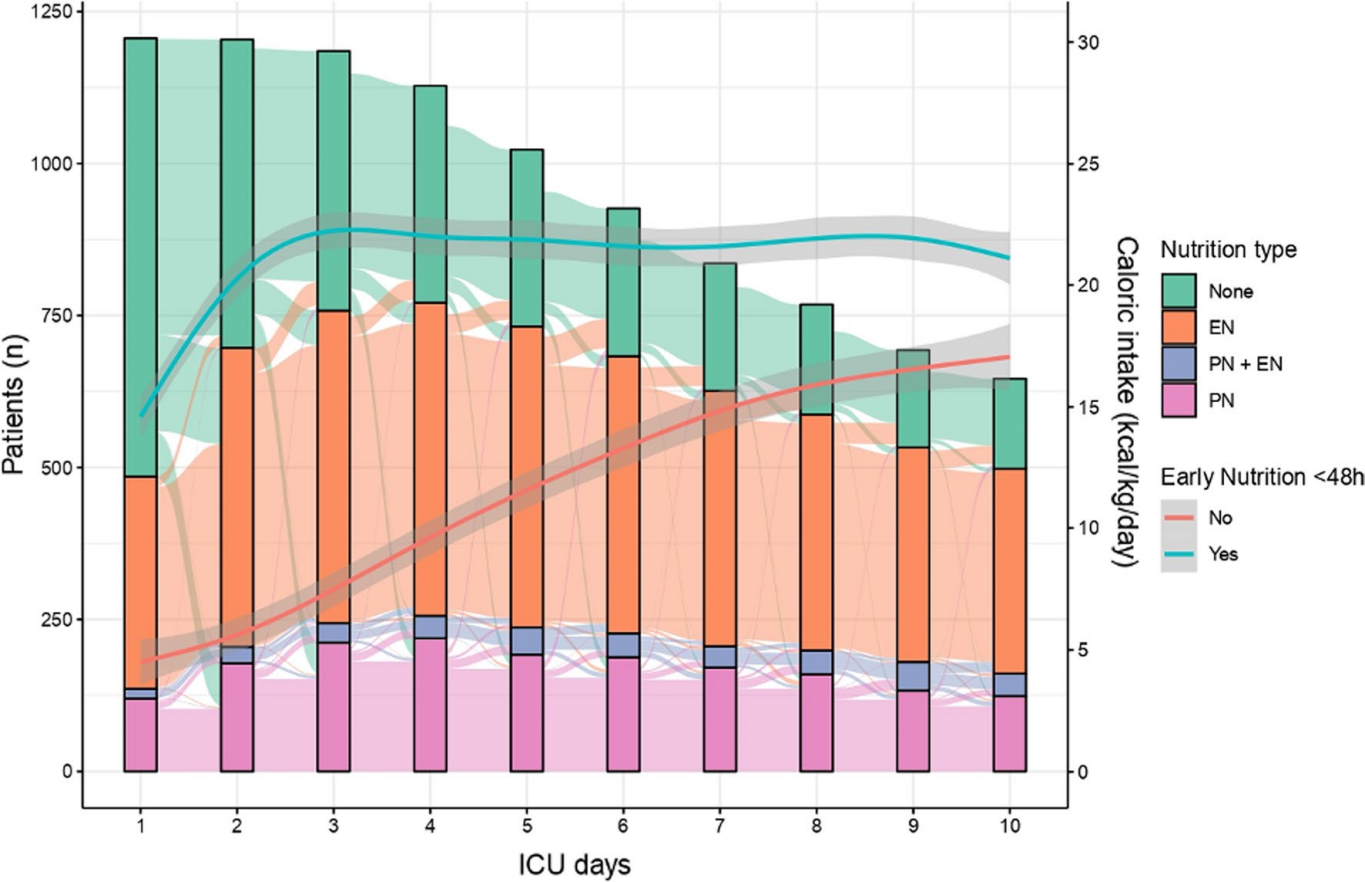
Ten-day evolution of early nutrition type and caloric intake. Alluvial plot showing the trend for the distribution of the different nutrition types per day for the 10-day follow-up period (left *y*-axis represents the number of patients). Introduction of nutrition support increased gradually from day 1 to day 10; enteral nutrition was the predominant route. Almost half of the patients received no nutrition support during the first two days. The peak number of patients receiving parenteral nutrition was observed on D4. Mixed nutrition (EN + PN) remained in the minority during the 10-day follow-up. The blue and red curves represent the energy intake trends for patients who received early nutrition support and those who did not, respectively (right *y*-axis expresses the amount of energy in calories per kilo per day). When early nutrition was administered, an energy intake plateau was reached on day 3 and remained stable thereafter. In the absence of early nutrition, intakes increased linearly and progressively over the 10 days. *EN* enteral nutrition, *EN* + *PN* simultaneous enteral and parenteral nutrition, *PN* parenteral nutrition, *ICU* intensive care unit

During the follow-up, 954 (79.1%) patients received nutrition support, with 753 (62.4%) patients receiving at least one day of enteral and 406 (33.7%) parenteral nutrition. Mixed nutrition (EN + PN) during the follow-up was provided to 158 patients (13.1%). The rate of delivery of nutrition support increased gradually from day 1 to day 10; enteral nutrition was the predominant route. The median timing of enteral introduction was at 38 [24–65] hours. The maximum number of patients receiving parenteral nutrition was observed on D4 of the ICU stay. When early nutrition was administered, the amount of energy provided increased until day 3 and remained stable thereafter. In the absence of early nutrition, intakes increased linearly and progressively over the follow-up period (Fig. 2). Daily energy and protein targets were reached at least once during the nutritional follow-up for 56% and 30% of all included patients, respectively. Gastrointestinal complications were frequent, with at least one occurrence of emesis or diarrhoea in 15.4% and 26.7% of the overall patients, respectively. Feeding intolerance occurred in 34% of ICU patients receiving enteral nutrition.

### Early nutrition and associated factors

Among the 718 (59.5%) patients who received early nutrition, initial enteral nutrition was administered to 504 patients (41.8%). Parenteral nutrition was the primary route in 214 patients (17.7%; including 35 patients who had mixed early EN + PN). Compared to those who received initial EN, patients receiving initial PN had higher 48-h energy (19.67 [14.30, 26.88] vs. 14.56 [9.78, 20.77]) and protein intakes (0.75 [0.45, 1.07] vs. 0.49 [0.27, 0.80]) (Additional file 3: Table S1). Overall early non-nutritional energy accounted for 3.16 [1.25, 5.63] kcal/kg/day.

Obese (aOR 0.71, 95% CI 0.52–0.97) and overweight patients (aOR 0.62, 95% CI 0.47–0.83) were less likely than patients with BMIs < 25 kg/m^2^ to receive early nutrition, whereas the SOFA score was positively associated with early nutrition support (aOR 1.07, 95% CI 1.04–1.1) (Additional file 3: Table S2).

A surgical diagnosis at admission was positively associated with the prescription of early parenteral nutrition (aOR 1.51, 95% CI 1.07–2.11) and negatively associated with enteral nutrition (aOR 0.7, 95% CI 0.53–0.92). Early invasive mechanical ventilation was significantly associated both with early enteral (aOR 9.84, 95% CI 6.54–14.81) and early parenteral nutrition (aOR 1.72, 95% CI 1.17–2.51). Early use of vasopressors was significantly associated with early enteral but not early parenteral nutrition (Additional file 3: Table S3).

Patients receiving early nutrition support by any route had significantly longer durations of invasive mechanical ventilation and longer ICU lengths of stay than patients who did not receive early nutrition (Table 2).

**Table 2:** Outcomes of patients with early nutrition and according to the type of early nutrition

	No early nutrition (*n* = 488)	Early enteral (*n* = 504)	Early parenteral (*n* = 214)	*P* value^†^
Duration of IMV (days)	4.0 [2.0, 10.0]	10.0 [5.0, 18.0]	7.00 [3.0, 13.0]	< 0.001
Duration of NIMV (days)	3.0 [2.0, 6.0]	3.0 [2.0, 4.0]	3.00 [2.0, 5.0]	0.017
Ventilator-free days D28 (days)	5.0 [2.0, 11.0]	3.0 [1.0, 8.0]	5.00 [2.0, 10.0]	< 0.001
ICU LOS	8.0 [5.0, 16.0]	13.5 [7.0, 24.0]	12.00 [7.0, 20.0]	< 0.001
28-day mortality	71 (14.6)	110 (21.9)	45 (21.2)	0.008
ICU mortality	61 (12.5)	119 (23.7)	43 (20.1)	< 0.001

Results are presented as the median (25th–75th percentiles) for quantitative variables and patient number (proportion) for qualitative variables. *N* = 1206

*IMV* invasive mechanical ventilation, *NIMV* noninvasive mechanical ventilation, *LOS* length of stay

^†^One-way ANOVA or Kruskal–Wallis between the three groups (no early, early enteral, early parenteral) according to the variable normality

### Association between initial nutrition support and patient mortality

Patients who died within 28 days were significantly older, had more frequently a medical admission diagnosis and had significantly higher initial SOFA scores. The proportion of non-survivors at D28 was increased among patients receiving early nutrition by any route, and among those receiving early enteral nutrition, compared to those without nutrition support. ICU survivors had significantly lower early energy and protein intakes (Table 1).

In the univariate analysis, early nutrition, by any route, was significantly associated with increased 28-day mortality. This association remained significant in the multivariable, multilevel analysis (aOR 1.56, 95% CI 1.11–2.2) and the propensity-weighted model (aOR 1.05, 95% CI 1.00–1.10, *p* = 0.031). Enteral nutrition was significantly associated with D28 mortality in the multilevel analysis (aOR 1.5, 95% CI 1.04–2.17), as well as in the propensity-weighted analysis (aOR 1.06, 95% CI 1.01–1.11, *p* = 0.03). Early parenteral nutrition, meanwhile, was associated with 28-day mortality in the multilevel analysis (aOR 1.72, 95% CI 1.07–2.77) but not in the propensity-weighted analysis (aOR 1.04, 95% CI 0.98–1.11, *p* = 0.203) (Table 3).

**Table 3:** Association between early nutrition and 28-day mortality

Variable	Crude* *N* = 1147		Multilevel analysis^†^ *N* = 1147		Propensity-weighted cohort^‡^ *N* = 1147	
	OR (95% CI)	*P*	aOR (95% CI)	*P*	aOR (95% CI)	*P*
*Binary early nutrition variable*						
Early nutrition						
No	1		1		1	
Yes	1.69 (1.23–2.34)	0.001	1.56 (1.11–2.2)	0.011	1.05 (1–1.1)	0.031
*Model 2 with early nutrition variable including nutrition type*						
Early nutrition						
None	1		1		1	
Enteral	1.7 (1.22–2.4)	0.002	1.5 (1.04–2.17)	0.031	1.06 (1.01–1.11)	0.03
Parenteral	1.66 (1.07–2.55)	0.021	1.72 (1.07–2.77)	0.027	1.04 (0.98–1.11)	0.203

*BMI* body mass index, *SOFA* Sequential Organ Failure Assessment

*Univariable logistic regression

^†^Multilevel, multivariable analysis with a random effect on the centre of inclusion and adjustment of age, sex, admission diagnosis type, BMI range and admission SOFA score

^‡^Propensity-weighted model with adjustment of age, sex, admission diagnosis type, BMI range and admission SOFA score

**Fig. 3:**
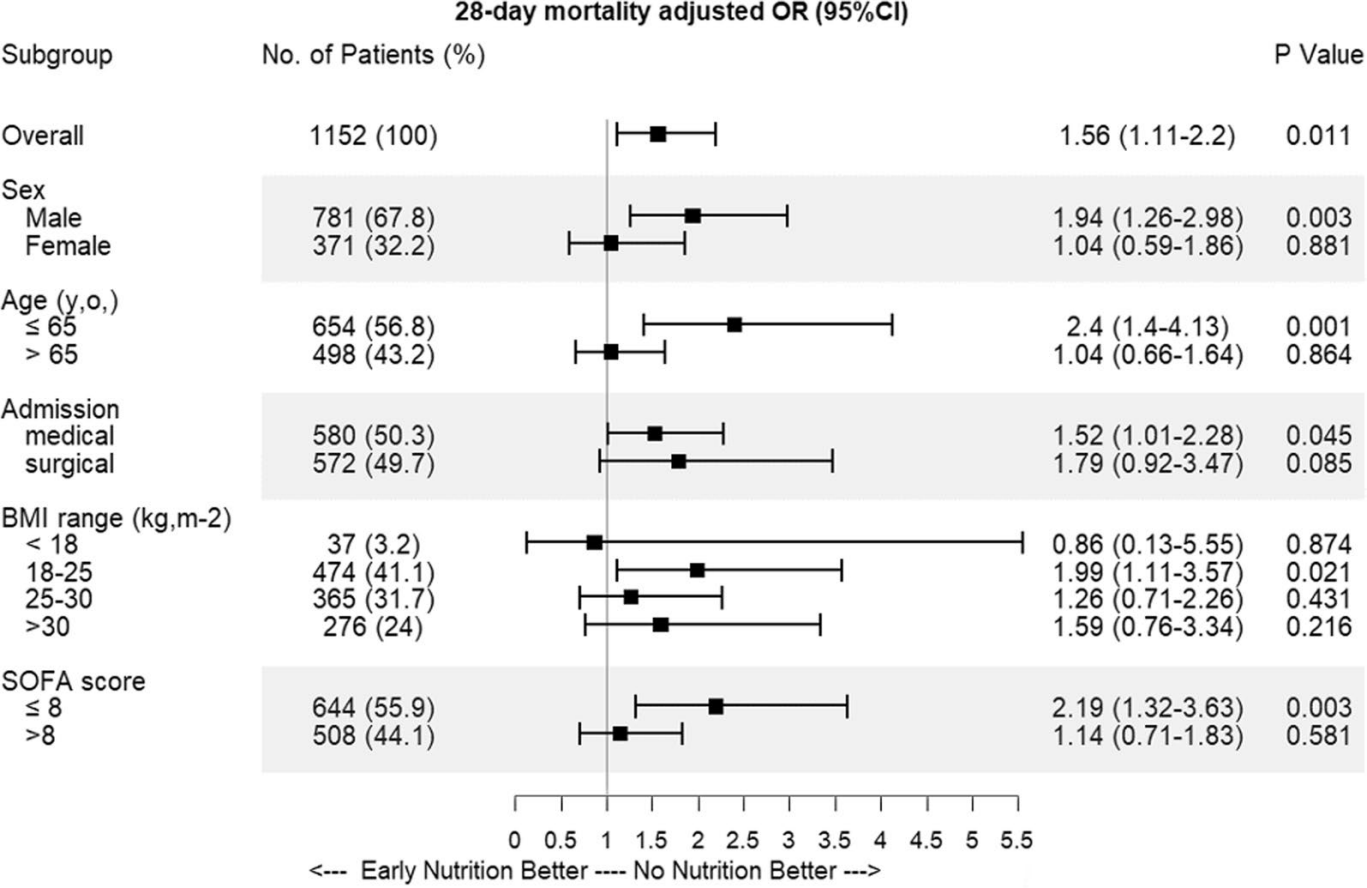
Early nutrition effects in different subgroups. Forest plot depicting the adjusted odds ratios (ORs) from a multilevel, multivariable analysis with a random effect on the centre of inclusion and adjustment of age, sex, admission diagnosis type, body mass index (BMI) range and admission Sequential Organ Failure Assessment (SOFA) score. The association between early nutrition and mortality at day 28 is assessed in subgroups according to sex, age, type of admission, BMI and SOFA score

In a subgroup analysis with the multilevel multivariable models, the association between early nutrition and 28-day mortality was strongest in male patients (aOR 1.94, 95% CI 1.26–2.98), those younger than 65 y.o. (aOR 2.4, 95% CI 1.4–4.13), with a BMI between 18 and 25 kg/m^2^ (aOR 1.99, 95% CI 1.11–3.57) or a SOFA score below 8 (aOR 2.19, 95% CI 1.32–3.63) (Fig. 3).

Regarding early macronutrient intakes, a significantly higher mortality risk was found in patients receiving any amount superior to 6 kcal/kg/d of energy and to 0.3 g/kg/d of protein. A potential dose-dependent effect was observed with early protein intake, with an increase in the mortality risk associated with increasing amount of protein/kg/day (aOR 1.43, 95% CI 1–2.05 for 0.3–0.9 g/kg/day and aOR 1.94, 95% CI 1.25–2.97 for > 0.9 g/kg/day) (Additional file 2: Fig. S2).

## Discussion

In this prospective observational study, almost two-thirds of the critically ill patients received early nutrition, primarily by the enteral route, as suggested by nutrition guidelines; however, energy and protein targets were met in only half and one-third of the patients, respectively. Early nutrition was more frequently observed in patients with multiple organ failure and less frequently in overweight and obese patients.

The administration of early nutrition by any route was significantly associated with increased 28-day mortality. This association was stronger in younger patients and those with less severe organ failure. Compared to patients who received no early nutrition, early enteral and parenteral nutrition appeared to be associated with increased mortality in the multivariable multilevel analysis; these associations remained significant in the propensity-weighted analysis for early enteral but not early parenteral nutrition. Situated within the literature, the four key findings of this study are as follows:

First, our study of nutrition practices revealed that more than three-quarters of the patients received nutrition support in the ICU and that their energy and especially protein intakes were below recommended targets. These results are consistent with those reported in recent nutrition trials [28]. A possible explanation might be the difficulties in reaching energy targets via the enteral route, chosen for 60% of our patients, with one-third suffering from feeding intolerance. Furthermore, the overall intakes may have been lower than in previous studies given that the reported volumes were the ones actually received by patients and not just the prescribed ones, even though we have taken into account the non-nutritional calories. Indeed, the difference between prescribed and actual intakes in the literature ranges from 10 to 30% due to digestive intolerance, airway management and organisational constraints [29, 30]. Regarding factors associated with early nutrition prescription, we observed that overweight and obese patients were less likely to receive early nutrition than patients with BMIs < 25 kg/m^2^. Similar results were previously found in an observational study reporting delayed nutrition support in obese critically ill patients [31]. This might result from the erroneous assumption that overweight and obese patients have sufficient resources to withstand the hypercatabolism associated with ICU stress. However, these patients may suffer from sarcopenic obesity at admission, which can worsen with protein malnutrition and ICU-acquired muscle weakness [32]. Recent data showing a protective effect of adipose tissue on sarcopenia attest to the complexity of energy metabolism in these patients and the need for further research [33–35].

Second, we found that early nutrition support is associated with day-28 mortality. This result could be explained by the risk of overnutrition during the acute phase of critical illness due to the uncontrollable endogenous energy produced by the stressed organism [36]. Indeed, the abrupt rise in intake in the ‘early nutrition’ group, which may be considered as early full-feeding, could then explain the deleterious effects observed. Moreover, in the absence of indirect calorimetry-based prescriptions, the risk of overnutrition in the acute phase is even higher, especially in patients treated with neuromuscular blocking agents, a therapy known to decrease basal metabolic rate. In contrast, after D4, Heidegger et al. demonstrated a beneficial effect of optimising the energy intake by individualising intakes using indirect calorimetry [37]. Despite a low level of evidence, generalisation of indirect calorimetry may help to estimate the intensity of basal metabolism after clinical stabilisation and to prevent the prescription of excessive amounts of energy [36, 38–40]. Furthermore, early nutrition could also inhibit the processes of autophagy—a survival mechanism that ensures the elimination of cellular waste and preservation of mitochondrial functions—and thereby limit the natural stress response to injury [41–43]. Finally, another explanation could be the occurrence of a refeeding syndrome, resulting from the premature introduction of high energy intakes in patients at risk. The latter hypothesis, although impossible to confirm in the absence of biological data in our cohort, seems plausible in view of two recent studies demonstrating the harmful effects of excessive and non-progressive intakes in patients with refeeding hypophosphatemia [44, 45]. Such findings, if confirmed in RCTs, could challenge our current practices on early nutrition in the ICU. In our study, patients not receiving nutrition support in the first 48 h had energy intakes from intravenous dextrose and propofol accounting for nearly 5 kcal/kg/day, close to the levels considered to be permissive underfeeding in recent RCTs [7, 46]. The negative effect of early nutrition seemed to prevail in younger patients and those with lower SOFA scores, which are characteristics of low nutritional risk in the NUTRIC score developed by Heyland et al. [47]. Early nutrition in patients at low nutritional risk (low NUTRIC score) exposes them to the risk of overfeeding and increased morbi-mortality. Our results are in conflict with those of Ortiz-Reyes et al. who found an association between early enteral and a reduced risk of persistent organ dysfunction plus day-28 death in subgroups of patients with a NUTRIC score < 5 or a SOFA < 9 [18]. Beyond obvious differences in study design (nested cohort analysis of an ongoing registry-based RCT) and intervention (early enteral alone), the reasons for these conflicting results may lie in the study population, which was predominantly of medical admission (82%) in this study. Furthermore, no significant association was found between different energy target and mortality in subgroups of NUTRIC score in a post hoc analysis of the PERMIT trial [48]. These data reinforce the need for individualisation of intakes and the need for clinical signs and accurate biomarkers to predict the optimal timing at which the body is able to metabolise external nutrients [49].

Third, we observed increased 28-day mortality for any early macronutrient doses higher than 6 kcal/kg/d of energy and 0.3 g/kg/d of protein. In a post hoc analysis of the EPaNIC trial, Casaer et al. demonstrated that an early low dose of macronutrients was associated with the fastest recovery compared to any higher dose, whether administered parenterally or enterally [50]. This finding is in line with the recent update of the ASPEN guidelines suggesting a lower energy target, between 12 and 20 kcal/kg/d, during the first week of the ICU [11]. A similar observation was made by Servia-Goixart et al. in a multicentre prospective study that included 639 critically ill patients, where a multivariable analysis adjusted for patient characteristics and severity showed that a higher mean energy intake was associated with mortality [51]. However, the authors reported a protective effect associated with the mean protein intake that did not match our results. A possible reason for these divergent findings may be that the protein intake was explored overall during the ICU stay, rather than focusing on the early phase as we did. Yet, early acute-illness-associated stress impairs protein–energy metabolism and its responsiveness to exogenous nutrients [52, 53]. Koekkoek et al. reported, in a recent retrospective study, a time-dependent association of protein intake with mortality: before day 3 in the ICU, a protein intake superior to 0.8 g/kg/day was associated with significantly higher 6-month mortality; after D3, a progressive increase protein provision was associated with an improvement in patients outcomes [54]. Compared to other macronutrients such as glucose and lipids, early administration of amino acids was associated with poor outcomes in a preplanned post hoc analysis of the PEPaNIC study [55]. The soon-to-be-published NUTRIREA-3 trial evaluating early low-energy, low-protein versus standard feeding in severe ICU-ventilated shock patients will add further light on these results [56].

Fourth, enteral nutrition was the most frequent route used to administer early nutrition support. We observed that early nutrition, through enteral and parenteral routes, was associated with higher mortality compared to no early nutrition; however, this effect was not found with early parenteral nutrition in our propensity-weighted analysis. In view of the small difference in OR for 28-day mortality between early enteral and early parenteral nutrition, it is essential to consider that this result may have stemmed from a lack of power due to the small number of patients receiving early parenteral nutrition in our study. Our results should not be misinterpreted as that parenteral nutrition is safe in high dosage and enteral is not. Progressive increase in energy should apply to both routes. The difference we observed between enteral and parenteral only encourages even more vigilance when prescribing early enteral. This association between mortality and early enteral nutrition is in contrast with the findings of recent studies. Indeed, the NUTRIREA-2 study did not find a significant difference in terms of mortality between early enteral and parenteral nutrition in ventilated patients with shock; however, the authors reported a higher incidence of bowel ischaemia and acute colonic pseudo-obstruction in the enteral group [19, 20]. Similarly, in our cohort, enteral intakes may have been pushed too quickly in the 'early nutrition' group, which may explain the detrimental association reported [57]. Pending additional data, early enteral nutrition administration should be thoroughly assessed and monitored in critically ill patients.

The strengths of our investigation include the large number of critically ill patients included in this European, binational, prospective observational study with detailed data on nutritional practices. Moreover, the study population shares similar demographic and clinical characteristics with another large, recent multicentric cohort of ICU patients; this supports the external validity of our observations [58]. Furthermore, the significant number of academic and non-academic participating centres provided a large panel of ICU patients, especially concerning their diagnoses of admission to the ICU. In contrast to registry-based studies, we reported the intakes actually received, not the prescribed ones, as well as the non-nutritional energy intakes. This precision in the acquisition of actual intakes allowed us to estimate, in the most precise manner, the level of macronutrients associated with a poor outcome. In addition, we presented several robust statistical approaches, propensity score weighting and multilevel models, to avoid potential indication bias or centre effect bias, frequently encountered in observational studies.

Nonetheless, the present study has several limitations. First, oral intake data were not collected due to the complexity of accurately estimating the energy intake from each food tray [59, 60]. This lack of data collection may only have led to the underestimation of the energy intake in a few patients given the high invasive mechanical ventilation rate and high severity scores we reported in our cohort. The oral route is barely proposed or used during the acute phase in critically ill patients due to well-known barriers including loss of appetite, dysphagia and general weakness [59, 61]. Second, we lacked the necessary data to estimate the nutritional risk using the Nutrition Risk Screening 2002 or the NUTRIC scores due to its limited use in clinical practice at the time of the survey [47, 62]. However, the report of such score may not have change our results considering recent study from Lew et al., which observed that the association between nutritional adequacy and 28-day mortality was independent of nutritional status [63]. Third, apart from emesis and diarrhoea, no other enteral-nutrition-related adverse events were reported, nor were the management strategies for feeding intolerance. Since the collection of these data, major advances have allowed clinicians to better characterise the severity of these complications using the Gastrointestinal Dysfunction Score and improve their management [64–66]. Fourth, we did not collect data on the staff involved in nutrition care. The presence of critical care dieticians in the ICU is known to be associated with significant improvements in macronutrient provision; however, their integration and precise role remain heterogeneous among units [67, 68]. Fifth, patients’ nutritional follow-up was limited to 10 days and outcomes to day-28. A growing number of articles in the literature insists on the relevance of nutritional support beyond the acute phase and the ICU stay [69, 70]. The definition of core outcomes for ICU nutritional trials now makes it possible to guide the choice of outcomes and to facilitate the comparison and interpretation of results [71]. Last, we did not report the energy requirements estimated by indirect calorimetry for our cohort, though this is recommended by the latest European Guidelines [14]. This choice was made to maximise the inclusion rate given the limited access to this technology in a significant proportion of participating centres.

## Conclusions

In this prospective cohort study including 1206 critically ill patients, nutrition support was widely prescribed, mostly by the enteral route. The administration of early nutrition was associated with higher day-28 mortality in a propensity-weighted logistic regression analysis, particularly for the enteral route.

These observations suggest that early provision of high amounts of macronutrients during the ICU stay may be associated with poor outcomes. These data are in contrast with current recommendations, based on low-quality evidence, on early enteral nutrition in the absence of a contraindication. They may inform future RCTs aimed at finding the optimal timing and amount of extrinsic macronutrients during the first days of the ICU stay. Aware of the variability of nutritional practices throughout the hospital stay, future work should also focus on the post-ICU phase to optimise the overall patient pathway and rehabilitation. The ongoing INTENT trial evaluating a whole-hospital nutrition intervention, not limited to the ICU, will provide essential data to shape our future nutritional practices [72].

### Supplementary Information

**Additional file 1. Fig. S1**: Propensity score balance. Comparisons of the absolute standardised mean differences (ASMDs) between the groups receiving early nutrition or not on selected covariates (age, sex, type of admission, BMI range and SOFA score at admission), before and after weighting. After propensity score weighting, the maximum ASMD decreases for all chosen covariates. The statistically significant difference between groups on each covariate is indicated by the solid circle. No significant difference persists after weighting. Standardised effects of less than 0.20 are considered low (better balance), 0.40 as moderate and 0.60 as large. A. Propensity score for the binary variable of early nutrition (yes/no). B. Multinomial propensity score for our three-factor variable (none/EN/PN).

**Additional file 2. Fig. S2**: Dose-dependent effect of early nutrition. Forest plots presenting the association between increasing the doses of calories (Figure 2A) and protein (Figure 2B) administered during the first 48 hours of the ICU stay and the mortality at 28 days. Adjusted odds ratios (aORs) were calculated using a multivariable logistic regression adjusted for age, sex, admission diagnosis type, BMI range and admission SOFA score; N=1147.

**Additional file 3. Table S1**: Characteristics according to early nutrition type. **Table S2**: Multivariable logistic analysis of factors associated with the administration of early nutrition support by any route (<48h). **Table S3**: Multivariable multinomial analysis of factors associated with the type of early nutrition.

## Data Availability

The datasets used and analysed during the current study are available from the corresponding author on reasonable request.

## Abbreviations

aOR: Adjusted odds ratio
APACHE II: Acute Physiology and Chronic Health Evaluation II
BMI: Body mass index
EN: Enteral nutrition
FRANS: French-Speaking ICU Nutritional Survey
ICU: Intensive care unit
IMV: Invasive mechanical ventilation
NIMV: Noninvasive mechanical ventilation
NMBA: Neuromuscular blocking agents
NUTRIC: Nutrition risk in critically ill
PN: Parenteral nutrition
RCT: Randomised control trial
SAPS II: Simplified Acute Physiology Score II
SOFA: Sequential Organ Failure Assessment
