# Supplementary figures and images for: Association between early nutrition support and 28-day mortality in critically ill patients: the FRANS prospective nutrition cohort study
Source: Crit Care. 2023 Jan 7;27:7. doi: 10.1186/s13054-022-04298-1 (PMC9826592; doi:10.1186/s13054-022-04298-1)

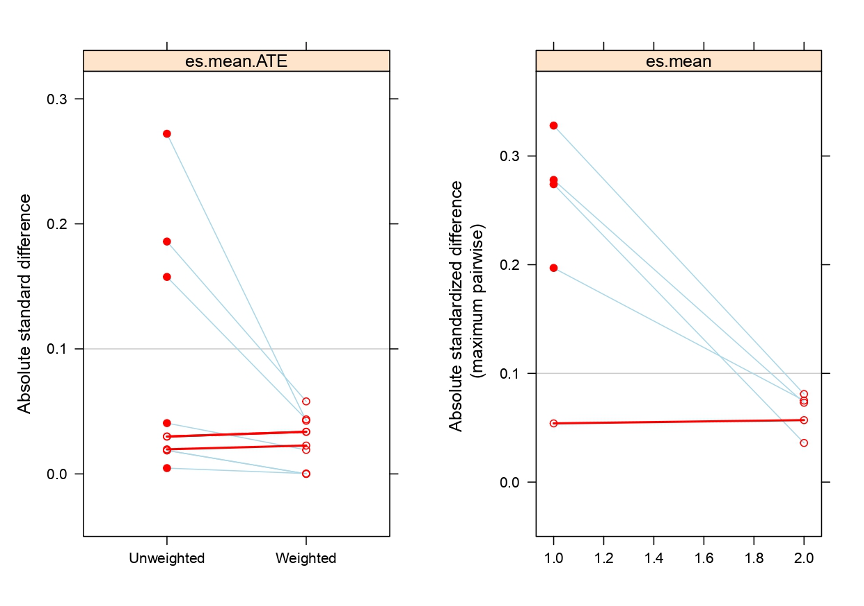

Supplement: Supplementary file 1 — Additional file 1. Fig. S1: Propensity score balance. Comparisons of the absolute standardised mean differences (ASMDs) between the groups receiving early nutrition or not on selected covariates (age, sex, type of admission, BMI range and SOFA score at admission), before and after weighting. After propensity score weighting, the maximum ASMD decreases for all chosen covariates. The statistically significant difference between groups on each covariate is indicated by the solid circle. No significant difference persists after weighting. Standardised effects of less than 0.20 are considered low (better balance), 0.40 as moderate and 0.60 as large. A. Propensity score for the binary variable of early nutrition (yes/no). B. Multinomial propensity score for our three-factor variable (none/EN/PN). [file 13054_2022_4298_MOESM1_ESM.png]

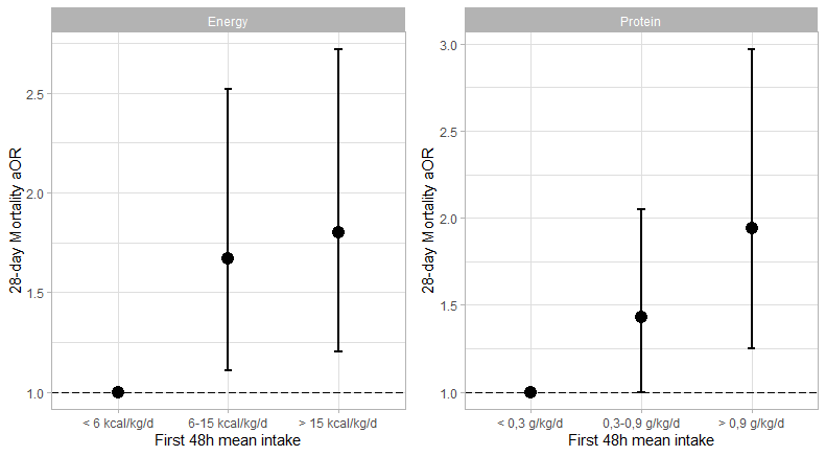

Supplement: Supplementary file 2 — Additional file 2. Fig. S2: Dose-dependent effect of early nutrition. Forest plots presenting the association between increasing the doses of calories (Figure 2A) and protein (Figure 2B) administered during the first 48 hours of the ICU stay and the mortality at 28 days. Adjusted odds ratios (aORs) were calculated using a multivariable logistic regression adjusted for age, sex, admission diagnosis type, BMI range and admission SOFA score; N=1147. [file 13054_2022_4298_MOESM2_ESM.png]
