# Supplementary material for: Association between early nutrition support and 28-day mortality in critically ill patients: the FRANS prospective nutrition cohort study
Source: Crit Care. 2023 Jan 7;27:7. doi: 10.1186/s13054-022-04298-1 (PMC9826592; doi:10.1186/s13054-022-04298-1)
Supplement: Supplementary file 3 — Additional file 3. Table S1: Characteristics according to early nutrition type. Table S2: Multivariable logistic analysis of factors associated with the administration of early nutrition support by any route (<48h). Table S3: Multivariable multinomial analysis of factors associated with the type of early nutrition. [file 13054_2022_4298_MOESM3_ESM.docx]

# Supplemental Figures and Tables

**sFigure 1. Propensity score balance.** Comparisons of the absolute standardised mean differences (ASMDs) between the groups receiving early nutrition or not on selected covariates (age, sex, type of admission, BMI range and SOFA score at admission), before and after weighting. After propensity score weighting, the maximum ASMD decreases for all chosen covariates. The statistically significant difference between groups on each covariate is indicated by the solid circle. No significant difference persists after weighting. Standardised effects of less than 0.20 are considered low (better balance), 0.40 as moderate and 0.60 as large. A. Propensity score for the binary variable of early nutrition (yes/no). B. Multinomial propensity score for our three-factor variable (none/EN/PN).

**sFigure 2. Dose-dependent effect of early nutrition.** Forest plots presenting the association between increasing the doses of calories (Figure 2A) and protein (Figure 2B) administered during the first 48 hours of the ICU stay and the mortality at 28 days. Adjusted odds ratios (aORs) were calculated using a multivariable logistic regression adjusted for age, sex, admission diagnosis type, BMI range and admission SOFA score; N=1147.

**sTable1. Characteristics according to early nutrition type.** Results are presented as the median (25^th^–75^th^ percentiles) for quantitative variables and patient number (percentage proportion) for qualitative variables. *BMI: body mass index, SAPS: Simplified Acute Physiology Score, SOFA: Sequential Organ Failure Assessment, APACHE: Acute Physiology and Chronic Health Evaluation, NMBA: neuromuscular blocking agents, IMV: invasive mechanical ventilation, NIMV: noninvasive mechanical ventilation, EN: enteral nutrition, PN: parenteral nutrition.*

|  |  | No early nutrition (n=488) | Early enteral (n=504) | Early parenteral (n=214) | P-value |
| --- | --- | --- | --- | --- | --- |
| Patient admission characteristics | | | | | |
| Age (years) |  | 61.9 [50.8, 72.6] | 62.1 [50.4, 72.0] | 65.7 [56.5, 74.9] | 0.005 |
| Height (cm) |  | 170.0 [165.0, 175.0] | 170.0 [165.0, 177.0] | 170.0 [162.0, 177.0] | 0.301 |
| Weight (kg) |  | 75.0 [65.0, 86.3] | 75.0 [64.0, 87.0] | 75.0 [63.0, 86.0] | 0.695 |
| Sex (%) | Female | 162 (33.2) | 163 (32.3) | 68 (31.8) | 0.923 |
|  | Male | 326 (66.8) | 341 (67.7) | 146 (68.2) |  |
| Admission type (%) | Surgical | 246 (50.4) | 230 (45.6) | 127 (59.3) | 0.003 |
|  | Medical | 242 (49.6) | 274 (54.4) | 87 (40.7) |  |
| BMI (kg/m^2^) |  | 26.1 [22.9, 29.7] | 25.2 [22.5, 29.4] | 25.6 [22.6, 29.7] | 0.241 |
| BMI range (%) | <18 | 15 (3.2) | 16 (3.2) | 6 (3.0) | 0.097 |
|  | 18–25 | 166 (35.8) | 224 (45.3) | 87 (43.1) |  |
|  | 25–30 | 168 (36.2) | 140 (28.3) | 61 (30.2) |  |
|  | >30 | 115 (24.8) | 114 (23.1) | 48 (23.8) |  |
| Country (%) | France | 397 (81.4) | 399 (79.2) | 207 (96.7) | <0.001 |
|  | Belgium | 91 (18.6) | 105 (20.8) | 7 (3.3) |  |
| University hospital (%) | Yes | 433 (88.7) | 427 (84.7) | 200 (93.5) | 0.003 |
|  | No | 55 (11.3) | 77 (15.3) | 14 (6.5) |  |
| Severity and organ support | | | | | |
| Admission SAPS II score | | 41.0 [31.0, 53.0] | 47.0 [36.0, 60.0] | 44.0 [32.5, 57.0] | <0.001 |
| Admission SOFA score | | 7.0 [4.0, 10.0] | 9.0 [6.0, 11.0] | 8.0 [4.0, 11.0] | <0.001 |
| Admission APACHE II score | | 18.0 [12.0, 23.0] | 20.0 [15.0, 25.0] | 18.0 [12.0, 24.0] | <0.001 |
| Early vasopressors (%) | | 242 (49.8) | 381 (75.6) | 131 (61.2) | <0.001 |
| Early IMV (%) |  | 258 (53.1) | 467 (92.8) | 145 (67.8) | <0.001 |
| Sedation (%) |  | 273 (55.9) | 423 (83.9) | 148 (69.2) | <0.001 |
| NMBA (%) |  | 59 (12.1) | 120 (23.8) | 37 (17.3) | <0.001 |
| Vasopressors (%) |  | 272 (55.7) | 399 (79.2) | 147 (68.7) | <0.001 |
| IMV (%) |  | 333 (68.2) | 466 (92.5) | 180 (84.1) | <0.001 |
| NIMV (%) |  | 206 (42.2) | 126 (25.0) | 76 (35.5) | <0.001 |
| Early nutritional intake | | | | | |
| Total Early Caloric intake (kcal/kg/day) | | 4.30 [2.36, 6.43] | 14.56 [9.78, 20.77] | 19.67 [14.30, 26.88] | <0.001 |
| Early non nutritional calories (kcal/kg/day) | | 4.30 [2.36, 6.43] | 2.82 [1.05, 5.33] | 1.69 [0.60, 4.18] | <0.001 |
| Early protein (g/kg/day) | | 0.00 [0.00, 0.00] | 0.49 [0.27, 0.80] | 0.75 [0.45, 1.07] | <0.001 |
| 10-day nutritional intake and complications | | | | | |
| Enteral nutrition (%) | | 150 (30.7) | 504 (100.0) | 99 (46.3) | <0.001 |
| Parenteral nutrition (%) | | 141 (28.9) | 51 (10.1) | 214 (100.0) | <0.001 |
| Total Caloric intake (kcal/kg/day) | | 6.42 [2.91, 14.91] | 19.70 [14.01, 24.75] | 22.35 [17.92, 26.87] | <0.001 |
| Non nutritional calories (kcal/kg/day) | | 2.98 [1.60, 4.45] | 1.93 [0.98, 3.46] | 1.31 [0.59, 2.42] | <0.001 |
| Protein intake (g/kg/day) | | 0.00 [0.00, 0.51] | 0.77 [0.51, 1.04] | 0.89 [0.70, 1.13] | <0.001 |
| Diarrhoea (%) |  | 110 (22.5) | 144 (28.6) | 68 (31.8) | 0.018 |
| Bowel movement (%) | | 383 (78.5) | 419 (83.1) | 167 (78.0) | 0.118 |
| Emesis (%) |  | 67 (13.7) | 86 (17.1) | 33 (15.4) | 0.348 |
| Feeding intolerance (%) | | 47 (31.3) | 171 (33.9) | 38 (38.4) | 0.516 |

**sTable 2.** **Multivariable logistic analysis of factors associated with the administration of early nutrition support by any route (<48h).** N=1152. Model adjusted for age, sex, admission type, BMI range and admission SOFA score*. aOR: adjusted odds ratio, BMI: body mass index, SOFA: Sequential Organ Failure Assessment.*

| Variable |  | aOR (95%CI) | P-value |
| --- | --- | --- | --- |
| Age (years) |  | 1 (0.99–1.01) | 0.760 |
| Sex | Female | Ref |  |
|  | Male | 1.1 (0.85–1.43) | 0.458 |
| Admission type | Surgical | Ref |  |
|  | Medical | 1.01 (0.79–1.28) | 0.967 |
| BMI range | 18–25 | Ref |  |
|  | <18 | 0.85 (0.42–1.73) | 0.637 |
|  | 25–30 | 0.62 (0.47–0.83) | 0.001 |
|  | >30 | 0.71 (0.52–0.97) | 0.033 |
| Admission SOFA score | | 1.07 (1.04–1.1) | <0.001 |

**sTable 3.** **Multivariable multinomial analysis of factors associated with the type of early nutrition.** N=1152. Model adjusted for age, sex, admission type, early invasive mechanical ventilation and early vasopressor therapy. *aOR: adjusted odds ratio, IMV: invasive mechanical ventilation.*

| Reference: No early nutrition | | Early enteral nutrition | | Early parenteral nutrition | |
| --- | --- | --- | --- | --- | --- |
|  |  | aOR (95%CI) | P | aOR (95%CI) | P |
| Age (years) |  | 1 (0.99–1.01) | 0.531 | 1.02 (1.01–1.03) | 0.002 |
| Sex | Female | Ref |  | Ref |  |
|  | Male | 1.04 (0.77–1.39) | 0.813 | 1.09 (0.77–1.55) | 0.636 |
| Admission type | Medical | Ref |  | Ref |  |
|  | Surgical | 0.7 (0.53–0.92) | 0.012 | 1.51 (1.07–2.11) | 0.017 |
| Early IMV | | 9.84 (6.54–14.81) | <0.001 | 1.72 (1.17–2.51) | 0.006 |
| Early vasopressors | | 1.51 (1.11–2.07) | 0.009 | 1.21 (0.84–1.75) | 0.314 |
